# From Circulating Biomarkers to Polymorphic Variants: A Narrative Review of Challenges in Thrombophilia Evaluation

**DOI:** 10.3390/jcm14103448

**Published:** 2025-05-15

**Authors:** Giuseppe Miceli, Anna Maria Ciaccio, Antonino Tuttolomondo

**Affiliations:** 1 Department of Health Promotion, Mother and Child Care, Internal Medicine and Medical Specialties (ProMISE) Università degli Studi di Palermo, Piazza delle Cliniche 2, 90127 Palermo, Italy; 2 Internal Medicine and Stroke Care Ward, University Hospital, Policlinico “P. Giaccone”, 90100 Palermo, Italy

**Keywords:** thrombophilia, circulating biomarkers, genetic biomarkers, thrombosis, D-dimer, Factor V Leiden, prothrombin G20210A, risk stratification, guidelines, personalized medicine

## Abstract

Thrombophilia is characterized by a hypercoagulable state that predisposes individuals to venous and arterial thrombotic events, posing significant challenges for clinical evaluation and management. This narrative review critically examines the current landscape of thrombophilia testing, focusing on the utility and limitations of both circulating and genetic biomarkers. Circulating biomarkers—such as D-dimer, antithrombin, protein C, and protein S—offer dynamic insights into the coagulation process yet often suffer from low specificity in varied clinical settings. In contrast, genetic biomarkers, notably Factor V Leiden and the prothrombin G20210A mutation, provide stable risk stratification but are limited by their low prevalence in the general population. Emerging markers, including selectins, Factor VIII, Factor XI, neutrophil extracellular traps, and extracellular vesicles, are also discussed for their potential to refine thrombotic risk assessment. By integrating evidence-based guidelines from international health organizations, this review underscores the need for a personalized approach to thrombophilia evaluation that balances comprehensive risk assessment with the avoidance of over-testing. Such an approach is crucial for optimizing patient outcomes and informing the duration and intensity of anticoagulant therapy.

## 1. Introduction

Thrombophilia—from the Greek *θρομβοφιλία*, meaning blood clot (*θρομβο*, *thrombo*) and affinity (*φιλία*, philia)—is an alteration of the coagulation system leading to a hypercoagulable state, which results in an increased risk of thrombotic venous and arterial events [1].

The global burden of thrombotic disorders is substantial, with venous thromboembolism (VTE), encompassing deep vein thrombosis (DVT) and pulmonary embolism (PE), affecting an estimated 1 to 2 per 1000 individuals annually in Europe and the USA [2]. Recurrent VTE represents a significant concern, with rates as high as 25% within five years after an initial event, underscoring the importance of comprehensive risk assessments [3].

**Figure 1:**
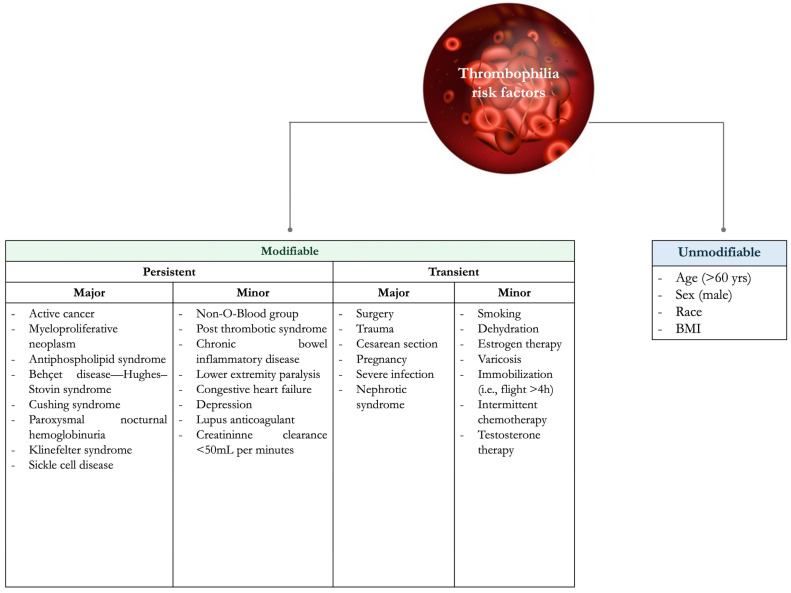
Risk factors for thrombophilia.

VTE risk factors can be classified as unmodifiable and modifiable (or acquired), which can be further divided into transient and persistent (Figure 1) [1].

In the mid-1800s, Rudolf Virchow pioneered the three pillars underpinning thrombophilia pathogenesis, which are still used to assess VTE risk: (i) venous stasis, (ii) vessel damage, and (iii) hypercoagulability [4]. The presence of at least two of these three factors increases a patient’s risk of developing DVT. Among the three, stasis is considered the most significant, and when it occurs alongside hypercoagulability or vessel damage, a clot typically forms. Furthermore, recent progress in molecular biology and a deeper understanding of pathophysiology have led to more advanced methodologies and an expanded concept of the triad, now including the roles of the endothelium, platelets, and soluble coagulation factors in thrombosis [4].

Thrombophilia poses significant clinical challenges in identifying at-risk individuals, diagnosing underlying etiologies, and tailoring effective therapeutic strategies. A thorough understanding of thrombophilia is critical, given its implications for recurrent VTE and other thrombotic complications. However, evaluating thrombophilia is inherently multifaceted, involving an array of circulating and genetic biomarkers. Circulating biomarkers, such as D-dimer, provide insights into the dynamic state of coagulation and fibrinolysis but often lack specificity or reliability in diverse clinical contexts. Conversely, genetic biomarkers, including the Factor V (FV) Leiden mutation and FIIG20210A, provide a more stable foundation for risk stratification. However, the low prevalence of thrombophilia genetic alterations does not support genetic screening in the general population. A concern commonly encountered in clinical practice is the inappropriate prescription of screening tests. An appropriate approach relies on a personalized thrombophilia evaluation of the patient’s characteristics, such as age and sex, personal and family history, clinical examination, and basic laboratory diagnostics. Integrating this information, the physician will decide whether the patient is a candidate for a circulating and genetic biomarker evaluation [5].

**Figure 2:**
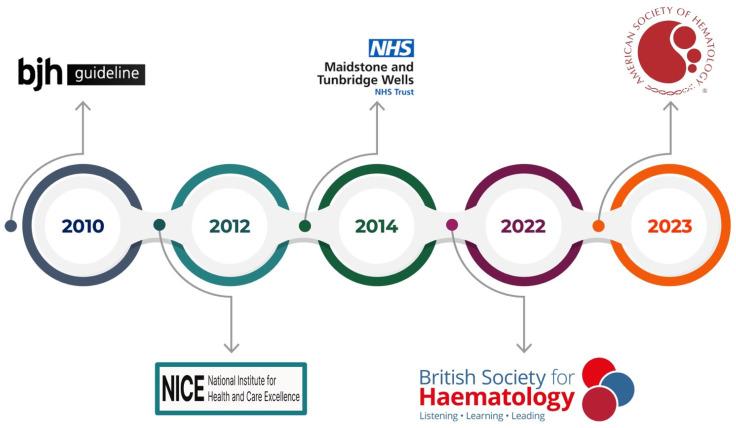
Guidelines on thrombophilia testing.

**Figure 3:**
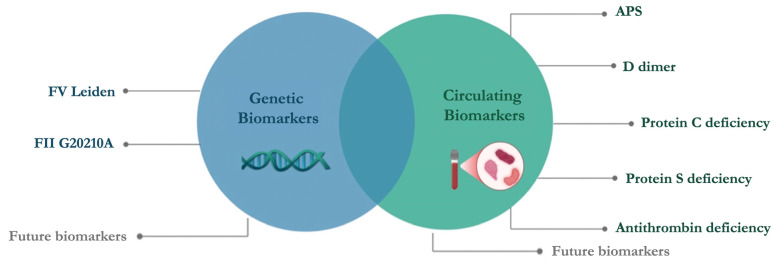
Circulating and genetic biomarkers of thrombophilia in clinical practice. APS, antiphospholipid syndrome.

This review aims to encompass the guidelines on thrombophilia testing (Figure 2) and provide an overview of the circulating and genetic biomarkers currently used in clinical practice (Figure 3). To date, manifold thrombophilia biomarkers have been investigated, with more than 1500 scientific articles published in PubMed. However, the international scientific community accepts only a few because of the lack of consistent evidence. Accordingly, this article focuses on selected established and emerging biomarkers of thrombophilia, emphasizing their clinical relevance, diagnostic challenges, and implications for patient care. This targeted approach aims to provide clinicians and researchers with a concise yet informative overview of key developments in the evolving landscape of thrombophilia diagnostics and management.

## 2. The Ideal Biomarker Identikit and Challenges in Thrombophilia

Over the past five decades, the definition of biomarkers has evolved and been continuously refined through scientific research and clinical advancements. The term “biomarker” was first introduced by Rho et al. in 1973 to describe the presence or absence of specific biological materials [6]. However, the concept dates back further, with Mundkur referring to “biochemical markers” in 1949 and Porter using the term “biological markers” in 1957 [7,8]. Since the early 1980s, the word “surrogate” has been used interchangeably with “biomarker”, derived from its literal meaning, “asked in place of”. A surrogate endpoint or marker is defined as a biomarker strongly associated with disease improvement [9].

Studies indicate a significant rise in the prevalence of the term “biomarker” compared to earlier terminology. The term itself is an abbreviation of “biological marker” and was formally defined by the National Institutes of Health (NIH) Biomarkers Definitions Working Group in 2000 [10]. According to this widely accepted definition, biomarkers serve as indicators of normal biological processes, pathogenic mechanisms, or pharmacological responses to therapeutic interventions.

Additionally, the U.S. Food and Drug Administration (FDA) defines a biomarker as a measurable indicator with potential applications across the entire disease spectrum, including research and therapy development, disease diagnosis and prognosis, monitoring disease progression, and assessing treatment responses. Collectively, a biomarker can be characterized as a specific component associated with normal biological functions, pathogenic processes, or responses to external factors or chemical agents, excluding the direct presence of the agent or its metabolites within body tissues [11,12]. In the Medicine of the Third Millennium, biomarkers are critical tools in assessing the health and disease status of individuals. Over the past decade, the discovery of biomarkers has exponentially increased. However, only a few of them have been introduced in clinical practice. Indeed, a biomarker is clinically relevant when it provides evidence to support a theoretically rational basis for use, such as the ability to reflect some measurement of, or change in, a physiologic or pathologic process or activity over a relatively short time. An ideal biomarker should have specific characteristics, including specificity allowing for an accurate differentiation between different physiological states; sensitivity for detecting even minimal changes in the biological system, ensuring the early identification of diseases or the response to treatment; noninvasiveness; clinical relevance, providing information that is directly applicable to patient care, such as aiding in diagnosis, prognosis, or monitoring therapeutic responses; and reproducibility and standardization, yielding consistent results across different laboratories and over time [13]. These attributes collectively ensure that a biomarker is reliable, practical, and valuable in enhancing patient outcomes. An optimal biomarker of thrombosis should accurately identify individuals predisposed to thrombosis before the occurrence of a clinical event; provide prognostic information for high-risk patients, such as those with cancer or atrial fibrillation; reflect ongoing thrombotic activity; respond to therapeutic interventions; and predict recurrent VTE. Overall, it should offer a clear benefit in guiding clinical decisions related to anticoagulation therapy and risk stratification. However, several challenges hinder the discovery and clinical implementation of an ideal thrombosis biomarker, such as the multifactorial nature of thrombosis, the lack of specificity, the variability in biomarker expression, and issues in standardization and reproducibility. Despite ongoing research, no single biomarker fully meets the criteria of an ideal thrombosis biomarker.

## 3. Thrombosis: Interaction Between Complex Systems

Thrombosis is a complex, multifaceted process involving the interplay of multiple biological systems with different players, including platelets, the endothelium, inflammatory pathways, and coagulation factors. These interactions give rise to the elements of Virchow’s triad, endothelial injury, abnormal blood flow, and hypercoagulability, which form the foundation of thrombus formation. However, due to the intricate and dynamic nature of these systems, identifying a single universal biomarker for thrombosis remains a challenge.

Platelets are among the first responders to vessel injury. They play a central role in thrombosis by adhering to the damaged endothelium and aggregating to form a platelet plug [14]. Upon activation, platelets release prothrombotic mediators, such as thromboxane A2, ADP, and serotonin, which enhance platelet aggregation and recruit additional platelets [15]. The expression of P-selectin and glycoprotein receptors facilitates interactions with leukocytes and coagulation proteins, further propagating thrombus growth.

The endothelium serves as both a barrier and an active modulator of coagulation [16]. Under physiological conditions, endothelial cells produce anticoagulant and fibrinolytic molecules, such as nitric oxide, prostacyclin, and tissue plasminogen activator (tPA) [17,18]. However, endothelial dysfunction, induced by inflammation, mechanical injury, or oxidative stress, promotes a prothrombotic state by expressing adhesion molecules (VCAM-1 and ICAM-1) and reducing anticoagulant signaling.

Inflammation is intimately linked to thrombosis through the cytokine-mediated activation of endothelial cells and platelets [19,20]. Pro-inflammatory cytokines, such as IL-1, IL-6, and TNF-α, upregulate tissue factor (TF) expression, triggering the coagulation cascade. Additionally, neutrophils release neutrophil extracellular traps (NETs), which serve as a scaffold for thrombus formation by trapping platelets and coagulation factors [21,22].

The coagulation cascade, composed of intrinsic and extrinsic pathways, culminates in the activation of thrombin, which converts fibrinogen to fibrin [23]. Fibrin serves as the structural framework of the thrombus, stabilizing platelet aggregates and incorporating red blood cells. The dysregulation of coagulation factors, whether due to genetic polymorphisms (e.g., Factor V Leiden) or acquired conditions (e.g., malignancy, antiphospholipid syndrome), significantly increases thrombosis risk.

Many different systems contribute to clot formation and each patient’s biology is unique. Thus, there is no one-size-fits-all biomarker for diagnosing or predicting thrombosis. For example, in some patients, platelet dysfunction may be the primary driver of thrombosis, whereas in others, excessive fibrin deposition or inflammation may play a dominant role. Additionally, the interactions between platelets, endothelial cells, inflammatory mediators, and coagulation factors are bidirectional and influenced by physiological conditions. The same molecule may exert prothrombotic or anticoagulant effects depending on the surrounding biochemical environment. Furthermore, genetic polymorphisms, comorbidities, and environmental factors contribute to significant variability in thrombotic risk. For example, individuals with prothrombotic mutations (e.g., Factor V Leiden and prothrombin G20210A) may exhibit heightened thrombotic responses compared to those without such mutations. Thus, due to the complexity and heterogeneity of thrombosis mechanisms, a multi-biomarker approach tailored to individual patient profiles is more viable than a one-size-fits-all model.

## 4. Guidelines on Thrombophilia Testing

Scientific societies and medical organizations worldwide have developed guidelines to support clinicians on the appropriate thrombophilia testing. These guidelines agree that testing should be selective and based on a patient’s history, rather than performed routinely for everyone who experiences a blood clot.

In 2010, Baglin et al. published the first clinical guidelines for thrombophilia testing [24]. Their recommendations stress that routine testing for heritable thrombophilia in unselected patients with venous thromboembolism (VTE) is not warranted. Instead, testing should be considered in specific scenarios, such as a first unprovoked VTE at a young age, a strong family history of VTE, or thrombosis occurring at unusual sites. Moreover, any test result should meaningfully influence patient management, whether by affecting the duration of the anticoagulation therapy or informing family planning. To investigate possible thrombophilic states, the guidelines recommend functional assays for antithrombin and protein C, immunoreactive assays for free protein S antigen, and an activated protein C (APC) resistance assay for Factor V Leiden. If the APC resistance assay is positive, the mutation should be confirmed via direct genetic testing, although beginning with a direct genetic test can eliminate the need for an APC resistance assay.

In 2012, the National Institute for Health and Clinical Excellence (NICE) released guidelines on managing suspected or confirmed VTE in adults, with updates in 2020 and 2023 [25,26]. These guidelines similarly discourage routine thrombophilia testing for all patients with VTE, recommending instead a targeted approach. Testing may be justified in patients with unprovoked VTE who have a first-degree relative with a history of VTE, in those with recurrent VTE, or in those presenting with VTE in uncommon sites. While the NICE emphasizes a structured methodology for VTE diagnosis and treatment, the guidelines do not specify the exact laboratory tests to perform. They do, however, recommend measuring D-dimer in low- or intermediate-risk patients to help rule out VTE.

Two years later, in 2014, the Maidstone and Tunbridge Wells NHS Trust issued guidelines reinforcing the principle of selective testing. Here, the focus is on ensuring that results directly influence clinical decisions and avoiding unnecessary anxiety or false reassurance. Thrombophilia testing is advised only for patients with a personal or family history of thrombosis in atypical settings, such as at a young age or with unprovoked events. Testing during acute thrombosis, while on anticoagulation medicine, or during pregnancy is generally discouraged unless results are urgently needed for management. Positive test outcomes should be handled with care to prevent unwarranted concern, and negative results must not overlook other potential risk factors. The recommended test panel comprises antithrombin, protein C, protein S, Factor V Leiden, and the FII G20210A mutation, with antiphospholipid antibodies as the only marker for acquired thrombophilia.

In 2022, the British Society for Hematology updated its guidance for evaluating both heritable and acquired thrombophilia [27]. Again, the overarching message is that testing should be context-dependent rather than routine. For provoked VTE, where a clear inciting factor is identified, testing for heritable thrombophilia typically does not alter management and is thus discouraged. However, in younger patients with unprovoked VTE or those with a significant family history, testing may guide decisions about the duration of anticoagulation therapy. In arterial thrombosis, heritable thrombophilia testing is generally unwarranted, given the weak association and limited impact on treatment. Testing for antiphospholipid syndrome (APS), however, is recommended for patients with arterial thrombosis who lack typical cardiovascular risk factors. Additionally, routine testing in unusual sites of thrombosis (e.g., splanchnic or cerebral veins) is not advised due to insufficient evidence linking these events to heritable thrombophilia. Screening asymptomatic individuals, including relatives of patients with VTE, is likewise discouraged because of the low absolute risk and the minimal effect on clinical management. For patients in whom thrombophilia testing is warranted, assessment typically includes Factor V Leiden and FII G20210A and evaluations for protein C, protein S, and antithrombin deficiencies. These deficiencies can arise from multiple genetic variants, so measuring plasma activities or concentrations remains the diagnostic mainstay. Testing physiologic anticoagulant levels is recommended after three months of anticoagulation for acute thrombosis. Even in asymptomatic first-degree relatives, testing is advised only if the results would change the management or influence major life choices.

In 2023, the American Society of Hematology (ASH) introduced guidelines for VTE management, offering evidence-based recommendations regarding when thrombophilia testing may help direct patient care. The ASH strongly advises against universal thrombophilia testing before initiating combined oral contraceptives (COCs), citing potential harms and costs that outweigh any benefits. In individuals with VTE attributed to significant transient (non-surgical) or hormonal risk factors, testing may be considered to guide the duration of anticoagulation therapy, although this is a conditional recommendation supported by low-certainty evidence. The ASH also suggests that in cases of cerebral or splanchnic venous thrombosis where discontinuing anticoagulation is under review, selective testing can inform treatment duration. Additionally, the ASH highlights how patients with a family history of antithrombin, protein C, or protein S deficiencies may benefit from testing, particularly when evaluating prophylaxis for minor provoking risk factors or the use of COCs or hormone replacement therapy. Testing for specific genetic polymorphisms, like MTHFR variants, is discouraged because they do not confer a significant increase in VTE risk. The ASH emphasizes that indiscriminate testing can lead to overdiagnosis, causing physical, psychological, and financial burdens without yielding tangible benefits. Clinicians are encouraged to engage in thorough discussions with patients before testing, outlining the test’s limitations and possible implications and how results may or may not influence management.

Over time, a clear consensus has emerged: indiscriminate thrombophilia testing is often unwarranted. Because most thrombotic events are multifactorial rather than purely linked to thrombophilia, routine panels can generate unnecessary costs, anxiety, and even mismanagement. In many cases, clinicians may order these tests reflexively, without considering whether outcomes will truly influence care decisions. Positive results can provoke undue alarm for patients and their families, while negative results may create a false sense of security and overlook other risk factors. Moreover, the substantial financial and logistical burden of testing underscores the need for a more judicious, individualized approach. A recent retrospective study by McRae et al. [28] reinforced these concerns, showing that in most instances, thrombophilia testing did not significantly alter treatment plans or patient management. Many tests were ordered under circumstances where results were unlikely to influence clinical decisions, indicating a need for more robust guidelines and better clinician education. Likewise, Anderson et al. observed that only 15% of patients with unprovoked VTE met appropriate criteria for thrombophilia screening, with no observed link between appropriate testing and either the detection of thrombophilia or the initiation of anticoagulation therapy. These findings collectively point to the overuse of thrombophilia testing and emphasize the importance of expert consultation when such testing is being considered. Overall, the evolving guidelines uniformly advocate a cautious, evidence-based strategy for thrombophilia testing—one that avoids unnecessary assays yet ensures that testing is available when it can meaningfully guide clinical management and optimize patient outcomes.

## 5. Biomarkers of Thrombophilia in Clinical Practice

Based on guidelines, the key circulating biomarkers used in clinical practice include D-dimer, PC, PS, AT, and antibodies to detect APS, while genetic biomarkers include only FV Leiden and FII G20210A (Figure 3).

### 5.1. Antiphospholipid Syndrome

APS is an acquired autoimmune disorder characterized by thrombosis and pregnancy complications due to the presence of antiphospholipid antibodies (aPLs) that interfere with normal blood clotting. APS is a major cause of acquired thrombophilia and can affect both arterial and venous circulation. It is rare in adults and extremely uncommon in children. The incidence and prevalence of APS vary geographically, with the U.S. having the highest prevalence, with an estimated incidence of 2.1 cases per 100,000 individuals, followed by Europe, at around 1.1 per 100,000, and Asia, where the incidence is even lower at 0.75 per 100,000 [29]. Low levels of aPLs can be detected in up to 10% of healthy individuals, and the prevalence of a positive aPL test increases with age. Additionally, the epidemiological variability is due partly to the heterogeneity testing. 

The pathophysiology of APS is not fully understood but is believed to result from a combination of genetic, environmental, and hormonal factors [30]. The key mechanism involves the production of aPLs, which target phospholipid-binding proteins, particularly beta-2-glycoprotein I (β2GPI). These antibodies can disrupt the clotting system, leading to excessive clotting; interfere with proteins like PC and PS; and promote a resistance to APC, making clots more likely. While they cause prolonged clotting times in laboratory tests, they also contribute to thrombosis in various organ systems. The most common sites for venous and arterial thrombosis are the lower limbs and cerebral arteries, respectively, although clot formation can occur in any organ. One major pathway linked to thrombosis in APS is Activated Protein C Resistance (APCR), which can be assessed using a thrombin generation assay. aPLs may impair APC function by interfering with FV activation by FXa or through direct interactions with FV on endothelial cells. Additionally, PC and PS, which are phospholipid-binding proteins, may also be affected by anti-PC and anti-PS antibodies, further contributing to APCR [31]. Platelets play a crucial role in APCR mediated by aPLs, as they influence APC sensitivity and serve as key cellular targets for aPLs [32]. Moreover, neutrophil extracellular traps (NETs) may interact with APC sensitivity and APCR in aPL-positive individuals. While these mechanisms provide insight into the APS pathogenesis, further research is needed to understand its complex pathophysiology fully.

Since 1999, APS diagnosis has been guided by the Sapporo classification criteria, later updated in Sydney [33]. However, these criteria have limitations, as APS symptoms extend beyond thrombosis and pregnancy complications. The classification does not account for non-criteria manifestations (e.g., livedo reticularis, cardiac valve damage, renal involvement, and thrombocytopenia) and lacks risk stratification based on biological profiles or cardiovascular risk factors. Patients with “triple positivity” (all three aPL tests are positive) have the highest thrombosis risk, which was not adequately addressed in previous criteria. To refine APS diagnosis, the American College of Rheumatology (ACR) and the European Alliance of Associations for Rheumatology (EULAR) initiated a revision in 2015 [34]. The updated ACR/EULAR classification criteria, published in August 2023, aim to be more precise and specific [35]. The 2023 ASH guidelines recommend against routine thrombophilia testing (hereditary or acquired) for patients with unprovoked VTE who have completed short-term anticoagulation. However, this approach may increase the risk of missing an APS diagnosis, potentially reducing opportunities to prevent arterial thrombosis and pregnancy complications [36]. Finally, in 2025, the ISTH-SSC Subcommittee on Lupus Anticoagulant/Antiphospholipid Antibodies released updated guidance on the laboratory detection and interpretation of aPLs for diagnosing APS. This document emphasizes the importance of the accurate testing and interpretation of aPLs, including anticardiolipin antibodies IgG or IgM, anti-β2GPI antibodies IgG or IgM, and Lupus anticoagulants [37]. Specifically, simultaneous testing for LA, aCL IgG and IgM, and aβ2GPI IgG and IgM is recommended. In clinical practice, detecting high-risk aPLA profiles, especially triple positivity (LAC, anti-β2GPI, and anticardiolipin antibodies), has significantly impacted management decisions. For instance, such patients are more likely to benefit from long-term anticoagulation with vitamin K antagonists than direct oral anticoagulants, which have shown inferior protection in this high-risk group [38].

The 2022 BSH guidelines recommend testing for aPLs after unprovoked VTE, since it may impact treatment decisions. Testing is also suggested in patients with VTE provoked by a minor risk factor, as it may influence antithrombotic therapy choices, and in patients with multiple acute thrombotic events and organ failure suggestive of catastrophic APS, the most severe form of APS, which is associated with high mortality [39,40]. On the other hand, family members of APS patients should not be screened, as APS is an acquired rather than hereditary thrombophilia.

The 2020 NICE guidelines suggest testing for aPLs in patients with unprovoked DVT or PE if anticoagulation therapy is being discontinued, though anticoagulants may affect test results, requiring specialist consultation. Finally, the BSH guidelines offer a balanced approach that could reduce APS misdiagnoses, thereby improving long-term thrombosis prevention.

### 5.2. D-Dimer

D-dimer is a fibrin degradation product released into the bloodstream when cross-linked fibrin is broken down by plasmin during fibrinolysis [41]. It has become a widely used biomarker in clinical practice for evaluating thrombotic disorders due to its high sensitivity for detecting active clot formation and breakdown. Increased levels indicate the presence of active thrombin generation and subsequent fibrin formation and degradation. In the context of thrombophilia, it is not a direct marker of a predisposition to thrombosis but rather a surrogate indicator of an ongoing or recent thrombotic event. The D-dimer evaluation provides important information to (i) rule out DVT or PE in low-risk individuals. Indeed, a normal D-dimer value effectively excludes VTE due to its high negative predictive value, which (ii) stratifies thrombophilia risk. Persistently elevated D-dimer levels in individuals without an acute event may suggest an ongoing hypercoagulable state. Additionally, it could be used to monitor thrombotic risk in patients with inherited or acquired thrombophilia, such as APS or FV Leiden, and to (iii) predict recurrent VTE, guiding the anticoagulation therapy duration, and (iv) assess thrombotic risk in specific populations, such as pregnant women [42].

Overall, although not thrombophilia-specific, increased D-dimer levels suggest a hypercoagulable state that can prompt further investigations into underlying thrombophilia conditions. D-dimer has the great advantage of being widely available, quick, and relatively inexpensive. Some limitations must be considered when evaluating D-dimer, including the low specificity. Indeed, its levels can increase in numerous conditions unrelated to thrombophilia, such as inflammation, infection, trauma, surgery, and advanced age [43]. Additionally, D-dimer reflects active clot formation and breakdown; thus, normal levels do not exclude thrombophilia in the absence of recent thrombotic activity. Normal physiological conditions, such as pregnancy, aging, and the postpartum period, can lead to an increase in D-dimer levels, misleading its interpretation.

D-dimer should be evaluated in patients with suspected thrombotic events, to monitor patients with known thrombophilic conditions, and as part of a broader evaluation for recurrent VTE risk following anticoagulation cessation.

D-dimer is a valuable biomarker for evaluating the active thrombotic processes and recurrent thrombosis risk in thrombophilia patients. While it lacks specificity and is not a direct marker of thrombophilia, its integration into comprehensive diagnostic and monitoring strategies offers significant clinical utility. D-dimer should be interpreted alongside other clinical and laboratory findings to maximize its effectiveness and be tailored to individual patient contexts.

D-dimer also guides clinical decision-making regarding anticoagulation duration. In the PROLONG study, patients with normal D-dimer levels one month after stopping anticoagulation for unprovoked VTE had a significantly lower recurrence rate, allowing for a safe discontinuation and reduced bleeding risk [44].

### 5.3. Antithrombin Deficiency

Antithrombin is a physiological natural anticoagulant. It belongs to the family of serine protease inhibitors (serpin), and it targets procoagulant serine proteases, such as activated FII, FIX, FX, and FXI, reducing clot formation. Heparin significantly enhances its anticoagulant function, induced by a conformational change that increases its affinity for target proteases. This mechanism underlies the therapeutic use of heparin in anticoagulation therapy. The deficiency or dysfunction of AT leads to an increased risk of thrombotic events, particularly VTE [45].

Among hereditary thrombophilia, AT deficiency is notable for its high thrombotic risk, with affected individuals experiencing a 50–85% lifetime risk of VTE. First described by Egeberg in 1965, AT deficiency has since been recognized as a critical factor in the pathogenesis of thrombotic disorders [46].

It is rare with an estimated prevalence in the general population ranging from 0.02% to 0.2%. Among individuals who experience venous thromboembolic events, such as VTE or PE, the prevalence is between 1% and 5%.

AT deficiency is classified into two types: (i) type I (quantitative deficiency), characterized by reduced levels of AT due to decreased synthesis, and (ii) type II (qualitative deficiency), caused by mutations affecting the functional ability of AT, despite normal levels. Type II can be further subdivided as follows: (i) type IIa (reactive site), due to mutations affecting the reactive site, impairing the inhibition of target proteases; (ii) type IIb (heparin-binding site), due to mutations reducing heparin affinity, diminishing the heparin-induced acceleration of AT activity; and (iii) type IIc (pleiotropic effects), due to mutations affecting multiple functional domains. AT deficiency is inherited in an autosomal dominant pattern with mutations primarily in the SERPINC1 gene located on chromosome 1q25.1. Homozygous mutations are typically incompatible with life, leading to severe thrombotic events in neonates. Thrombotic events often occur at a young age and may be recurrent. Pregnancy-related complications, such as recurrent miscarriages and intrauterine fetal demise, have also been associated with AT deficiency.

The diagnosis of an AT deficiency relies on both functional and antigenic assays. Functional assays measure the inhibitory activity of AT against target proteases, i.e., thrombin or FXa, while antigenic assays quantify the concentration of AT protein. A combination of these tests helps differentiate between type I and type II deficiencies. Laboratory testing guidelines for diagnosing antithrombin deficiency recommend starting with an activity assay. If activity levels are reduced, follow-up testing should include an antigen assay and the calculation of the activity-to-antigen ratio [47]. Pediatric reference ranges should be applied for children up to six months of age. It is essential to rule out acquired causes of reduced antithrombin levels, such as liver dysfunction, protein loss (e.g., via proteinuria), heparin therapy, disseminated intravascular coagulation, recent thrombosis, or surgery, as well as potential causes of falsely normal or elevated results, including the use of anticoagulants like argatroban, bivalirudin, and dabigatran in thrombin-based assays or rivaroxaban, apixaban, and edoxaban (but not betrixaban) in factor Xa-based assays. When available, molecular testing can aid in assessing thrombotic risk, which may vary depending on the specific genetic mutation, and can also detect variants that traditional activity assays may miss. Strategies for interpreting these laboratory findings are also provided.

The identification of hereditary antithrombin deficiency can significantly influence clinical management by prompting lifelong anticoagulation in individuals with a strong family history of thrombosis or recurrent unprovoked VTE. Furthermore, during high-risk situations, such as surgery or pregnancy, AT concentrate replacement therapy has been used to prevent thrombosis in deficient patients [48].

Antithrombin deficiency is a critical hereditary thrombophilia with significant implications for thrombotic risk. Recognizing its role as a biomarker enables targeted prevention and management strategies, ultimately improving patient outcomes. Ongoing research into the molecular mechanisms and therapeutic interventions continues to enhance our understanding and approach to this condition.

### 5.4. Protein C Deficiency

PC is a vitamin K–dependent zymogen that plays a critical role in regulating thrombosis and hemostasis. The protein was first purified and characterized by Stenflo in 1976 [49].

It is synthesized in hepatocytes and circulates as a heterodimeric complex composed of heavy and light chains. Its physiological activation occurs on the endothelial cell surface and requires two membrane receptors: the endothelial protein C receptor (EPCR) and thrombomodulin. These receptors are essential cofactors in the conversion of thrombin-mediated PC into activated PC (APC). The binding of thrombomodulin to thrombin enhances PC activation by three to four times, while EPCR binding to the zymogen further accelerates the activation rate by 20-fold.

Upon activation by thrombin bound to thrombomodulin, PC is cleaved and converted into activated PC. APC exerts its anticoagulant function by interacting with PS as a cofactor, inactivating key coagulation factors. Specifically, APC cleaves FVa at Arg534, Arg334, and Arg707, effectively downregulating thrombin generation. Additionally, APC inactivates FVIIIa by cleaving bonds at Arg355 and Arg581, with FV serving as an additional synergistic cofactor. These interactions are critical for maintaining hemostatic balance and preventing excessive clot formation.

Beyond its anticoagulant function, APC also exerts cytoprotective and anti-inflammatory effects. These properties are largely mediated by the EPCR-dependent cleavage of protease-activated receptor 1 (PAR1) on endothelial cells. Additionally, APC contributes indirectly to fibrinolysis by binding to plasminogen activator inhibitor-1 (PAI-1), which enhances the activity of tissue-type plasminogen activator (tPA). Moreover, since APC reduces thrombin generation by inactivating FVa and FVIIIa, the activation of thrombin-activatable fibrinolysis inhibitor (TAFI) is also reduced, further promoting profibrinolytic activity.

A deficiency in PC leads to a hypercoagulable state, increasing the risk of VTE, including DVT and PE. PC deficiency was first described by Griffin et al. in a family with a history of recurrent venous thrombosis and low PC antigen levels. Subsequent research established its association with thrombophilia. The clinical spectrum of PC deficiency varies from venous thromboembolism to acute, life-threatening complications such as purpura fulminans and disseminated intravascular coagulation (DIC). PC deficiency can be congenital or acquired. Congenital PC deficiency results from mutations in the PROC gene, located on chromosome 2q14.3. To date, over 160 distinct PROC mutations have been identified as causes of PC deficiency. It has been estimated that in patients with suspected hereditary VTE, the prevalence of PC deficiency is 5–10%. Among the general Caucasian population, the prevalence of PC deficiency is estimated to be 0.2–0.4% [50]. Acquired forms are often transient and reversible when the underlying cause is addressed. It occurs due to factors affecting protein C production, activation, or consumption, including malabsorption syndromes (e.g., celiac disease and Crohn’s disease), prolonged antibiotic use (disrupting gut microbiota that synthesize vitamin K), poor dietary intake (seen in malnutrition or chronic illness), or warfarin therapy (initially depletes protein C levels before reducing thrombin generation). Also, diseases affecting the liver, such as cirrhosis, hepatitis, or liver failure, impair its production.

There are two primary laboratory tests for detecting PC deficiency: antigen assays and activity assays (clot-based or chromogenic). Both tests are performed on sodium citrate (3.2%) plasma samples. The reference range for PC activity and antigen levels in adults typically falls between 70% and 140%, though exact values may vary by the assay and laboratory. The PC activity assay is the recommended screening test and can be either clot-based or chromogenic, depending on the assay design. A chromogenic assay is endorsed for its high specificity; however, it cannot detect the rare type 2b protein C deficiency, which involves impaired interactions with calcium ions, phospholipids, protein S, and coagulation factors Va and VIIIa. In contrast, the clotting-based assay can identify the type 2b deficiency but is limited by its lower specificity [51].

PC antigen assays measure the total quantity of PC but do not assess its function, making them unsuitable for detecting type II PC deficiencies (where PC is present but dysfunctional). These assays use monoclonal or polyclonal antibodies by the ELISA in most clinical laboratories. It is important to note that PC levels vary with age; therefore, adult reference ranges are not applicable to infants or children, and even during adolescence, PC levels may remain below adult values. Additionally, pre-analytical factors in the specimen can influence PC measurements and are often assay-dependent. For instance, a partially clotted sample may yield an artificially elevated PC level in a chromogenic assay but a falsely decreased level in a clotting-based assay. Finally, direct oral anticoagulants can falsely increase PC levels when measured by clotting-based assays, whereas standard chromogenic assays remain unaffected [51].

Since PC activity assays detect both quantitative and functional deficiencies, they remain the preferred screening test. However, none of the available PC activity assays can assess all functional aspects of the PC molecule, and many laboratories favor chromogenic activity assays for initial screening.

Detecting protein C deficiency in individuals with a personal or family history of VTE has led to preventive anticoagulation in high-risk periods (e.g., during long-haul travel, pregnancy, or post-surgery), thereby reducing VTE recurrence [52].

### 5.5. Protein S Deficiency

Protein S is a vitamin K-dependent glycoprotein that serves as a natural anticoagulant by enhancing the action of APC. First identified in Seattle, Washington, in 1979, its name reflects its place of discovery. A deficiency in PS disrupts normal coagulation control, leading to an increased risk of thrombophilia and VTE due to an excessive blood clot formation [53].

PS deficiency can be either congenital or acquired. The latter is more common than hereditary forms and can result from various conditions, including vitamin K deficiency, liver disease, DIC, nephrotic syndrome due to protein S loss, pregnancy, oral contraceptives, hormone replacement therapy, autoimmune disorders such as APS and systemic lupus erythematosus (SLE), because autoantibodies can bind to protein S, reducing its function. Finally, severe infections and inflammatory states can downregulate protein S expression. Mutations in the PROS1 gene are responsible for congenital PS deficiency [54,55]. The majority of PROS1 mutations are point mutations, including transversion mutations, which lead to the formation of a premature stop codon, resulting in a truncated, nonfunctional protein S molecule. Over 200 different PROS1 mutations have been identified, giving rise to distinct forms of PS deficiency. In particular, the International Society on Thrombosis and Hemostasis (ISTH) categorizes PS deficiency into three phenotypes based on the total protein S antigen, free protein S antigen, and protein S activity: (i) type 1 (quantitative deficiency), characterized by low levels of total protein S (TPS) and free protein S (FPS), along with reduced protein S activity; (ii) type 2 (functional deficiency, also known as type 2b), characterized by normal levels of TPS and FPS, but with decreased protein S activity, indicating a functional defect; and (iii) type 3 (selective free protein s deficiency, also known as type 2a), characterized by normal TPS levels but reduced FPS and PS activity, reflecting a quantitative defect affecting only the free, active form of protein S [56]. Congenital PS deficiency follows an autosomal dominant inheritance pattern. Heterozygous mutation typically exhibit a mild PS deficiency, whereas homozygous or compound heterozygous mutations result in severe PS deficiency, which can lead to life-threatening thrombotic complications. It has been reported that a heterozygous mutation typically leads to thrombosis before the age of 40–45, while a homozygous mutation presents with thrombotic events in early infancy and is often life-threatening.

Approximately 50% of heterozygous individuals develop VTE, while the remaining half remain asymptomatic throughout their lives. The estimated incidence of mild congenital PS deficiency is approximately 1 in 500 individuals, while severe PS deficiency is exceedingly rare, with an unknown prevalence due to diagnostic challenges [53].

PS deficiency is uncommon in individuals without a history of VTE. In a study of healthy blood donors, the prevalence of familial PS deficiency ranged between 0.03% and 0.13%. However, the prevalence increases significantly among patients with recurrent thrombosis or a family history of thrombosis, ranging from 3% to 5% [57].

Some studies suggest that adjusting the diagnostic cutoff for PS levels could impact the prevalence of the disorder, as lower thresholds might increase detection rates [58]. Data from the United States and Europe indicate a similar prevalence of protein S deficiency, whereas studies in the Japanese population show a higher frequency. Among Japanese patients with VTE, the prevalence of protein S deficiency is 12.7%, while it is estimated from 0.48% to 0.63% in the general Japanese population [59].

The diagnosis of PS deficiency relies on the measurement of PS antigen levels and functional activity. PS antigens can be detected as the total antigens or free protein S antigens. The free form is functionally active. Both free and total protein S can be measured by an ELISA. PS functional activity assays measure the biological activity of PS by assessing its ability to function as a cofactor for APC in the inactivation of FVa and FVIIIa. They include clot-based assays, which measure the ability of APC + PS to extend clotting time, and chromogenic protein activity assays, which measure PS activity by detecting its role in APC function using synthetic chromogenic substrates. Several factors can influence PS levels, making accurate diagnosis challenging. Thus, repeat testing in stable conditions is recommended. Specifically, vitamin K deficiency, liver disease, warfarin therapy, pregnancy and hormonal therapy, nephrotic syndrome, severe infections or inflammatory states (downregulation of protein S synthesis), and FV Leiden causes false low PS levels. Additionally, PS levels also fluctuate due to age, sex, hormonal influences, and lipid metabolism. Notably, women generally have lower total and free protein S levels than men. Total PS levels increase with age, particularly in women, due to hormonal variations, whereas free PS levels remain stable over time.

Total protein S tests are highly effective but do not detect type 2 and type 3 protein S deficiency. Free protein S assays can be an alternative, but their reproducibility is limited. Assessing APC cofactor activity may act as an indirect marker of protein S deficiency, though these assays tend to have a high false-positive rate.

If protein S deficiency is confirmed, genetic testing of the PROS1 gene can help confirm hereditary deficiency.

In conclusion, the PS deficiency testing approach relies on first measuring the PS activity assay, which represents the preferred screening test. If the activity is low, measure the total and free PS antibody levels. After ruling out acquired causes and if a hereditary deficiency is suspected, PROS1 genetic testing should be performed. PROS1 genotyping can be important in diagnosing PS deficiency, and the ISTH maintains a registry of documented mutations.

### 5.6. FV Leiden

In 1993, Swedish hematologist Björn Dahlbäck observed an unusual resistance to activated protein C (APC) in some families with a history of venous thrombosis [60]. Using an activated partial thromboplastin time (aPTT)-based assay, his team found that APC failed to prolong the clotting time as expected in certain individuals. This novel thrombophilic phenotype was termed Activated Protein C Resistance (APCR). Dahlbäck’s work revealed that APCR was significantly more prevalent among individuals with DVT compared to the general population, indicating a strong association with VTE.

In 1994, Pieter Bertina and colleagues identified the genetic basis of APCR as a point mutation in the FV gene: a guanine-to-adenine (G > A) substitution at nucleotide position 1691, resulting in the replacement of arginine by glutamine at amino acid position 506 (Arg506Gln) [61]. This historical numbering refers to the mature FV protein; however, readers should be aware that the American College of Medical Genetics and Genomics’ standardized nomenclature suggests the numbering of proteins according to their whole length regardless of post-translational maturation, i.e., the cleavage of pre-propeptides. Therefore, the numbering of amino acids starts at the first methionine, from the first translation codon. In case of FV Leiden, this corresponds to p.Arg534Gln. The Arg506Gln mutation impairs the normal inactivation of FVa by APC, leading to an increased thrombin generation and a hypercoagulable state. Because this mutation was first identified in Leiden, Netherlands, it became known as the FV Leiden mutation [62]. This variant accounts for over 90% of inherited APCR cases and remains the most common genetic risk factor for thrombophilia in individuals of European descent, with a population prevalence from 1% to 5% on average, which is even higher among patients with VTE (10–20%).

Heterozygosity for FV Leiden increases the lifetime risk of thrombosis by approximately 7-fold, while homozygosity, though rare, confers a roughly 20-fold increase in risk [63]. Despite this, heterozygosity does not appear to significantly affect overall mortality. The diagnosis of FV Leiden typically involves a two-step process: the initial detection of APC resistance through a functional clotting assay, followed by confirmatory genetic testing for the 1691G > A substitution.

Importantly, APC resistance can also occur in the absence of FV Leiden. Several non-FV Leiden inherited APC resistance variants have been described, including mutations that affect other cleavage sites of factor V or alter its cofactor activity with protein S. These rare variants can be functionally significant and may not be detected by genotyping for FV Leiden alone. Therefore, the functional APC resistance assay remains a critical tool for identifying both FV Leiden-associated and non-FV-Leiden-mediated APC resistance [64]. According to recent evidence [65,66], these assays play an essential role in comprehensive thrombophilia evaluation, particularly in patients with a strong clinical phenotype but negative genetic tests for common variants.

In clinical practice, the identification of APC resistance, particularly FV Leiden, has informed decisions around contraceptive use and pregnancy management. Women with this mutation are typically advised against estrogen-containing contraceptives and may be considered for prophylactic anticoagulation during high-risk periods, such as pregnancy, especially if additional risk factors are present [67].

### 5.7. FIIG20210A

Factor II, also known as prothrombin, is a glycoprotein synthesized in the liver. It is a vitamin K-dependent factor, with a pivotal role in the coagulation cascade. It is activated into thrombin by the prothrombinase complex, which consists of FXa, FVa, calcium ions, and phospholipids from platelet membranes. Once activated, thrombin (FIIa) facilitates blood clot formation by several mechanisms, including the conversion of fibrinogen into fibrin, which forms a stable clot; the activation of FXIII, which strengthens the fibrin clot; the activation of FV and FVIII, which accelerates clot formation; and the stimulation of platelets, which enhances clot stabilization.

The second most common cause of hereditary thrombophilia is the FII G20210A mutation occurring in the FII gene where guanine is replaced by adenine at position 20210 in the FII gene, resulting in an increased synthesis of FII [68]. Heterozygous carriers have a 2–3 times higher risk of blood clots, while homozygous individuals face a significantly higher clotting risk. The FII G20210A mutation is mostly found in individuals of European descent, with an estimated prevalence of 1% to 3% in the general population. It is rare in Asian, African, and Indigenous populations, with frequencies typically below 0.5%. Among individuals with VTE, the prevalence of Factor II G20210A is significantly higher (6% to 10%), compared to the general population. The mutation is more frequently observed in individuals with DVT and PE. The homozygosity is extremely rare (~1 in 5000 individuals) but associated with a significantly higher clotting risk (10–20 times greater than normal) [69].

Recent studies have further elucidated the clinical implications of the FII G20210A mutation. A recent study indicated that individuals with this mutation may experience a lower risk of major bleeding during extended anticoagulation therapy, suggesting a favorable risk–benefit profile for prolonged treatment in certain patients [70]. Additionally, the ASH’s 2023 guidelines emphasize the importance of individualized risk assessment when considering the duration of anticoagulation in patients with inherited thrombophilias, including the FII G20210A mutation. These insights support a more tailored approach to managing patients with this genetic variant, potentially improving outcomes by balancing the risks of thrombosis and bleeding.

### 5.8. Rare Genetic Thrombophilia States: Emerging Insights and Unidentified Risk Factors

While common hereditary thrombophilias, such as Factor V Leiden and prothrombin G20210A, are well characterized, a growing body of evidence points to rare genetic thrombophilia states that also contribute to VTE risk. These include emerging conditions such as antithrombin resistance and FIX Padua, among others. Furthermore, accumulating data from genomic and proteomic studies suggest that additional, yet unidentified genetic variants may contribute to unexplained thrombophilia.

AT resistance is an emerging and underrecognized form of thrombophilia. Traditionally, AT deficiency has been linked to decreased levels or dysfunctional antithrombin protein. However, recent studies have identified mutations in the prothrombin gene (F2 Arg596Leu, Arg596Gln, and Arg596Trp) that confer resistance to the inhibition by antithrombin, despite normal AT levels and activity assays [71]. These mutations result in a thrombin molecule that is functionally resistant to AT, leading to sustained thrombin activity and increased thrombotic risk. Affected individuals may have normal results in standard AT assays, and diagnosis relies on functional thrombin inhibition testing and genetic analysis.

FIX Padua is a rare gain-of-function mutation in the F9 gene (Arg338Leu), resulting in elevated factor IX (FIX) activity [72]. Originally discovered in association with thrombosis in a family with elevated FIX levels, this mutation increases the coagulant potential without affecting FIX antigen levels. Carriers of FIX Padua exhibit hypercoagulability due to the enhanced activation of the coagulation cascade. FIX Padua has also been repurposed in gene therapy vectors for hemophilia B due to its high specific activity.

Several other rare or recently identified mutations may contribute to inherited thrombophilia, including PC or PS dysfunctional variants, which are associated with functionally impaired proteins without quantitative deficiency; FV Cambridge and Liverpool variants, which confer APC resistance; and gain-of-function mutations in factors VII, X, and XI, which have been linked to familial VTE in rare reports but require further validation.

Finally, epigenetic or regulatory mutations in non-coding regions or in transcriptional regulators of coagulation genes may alter expression levels.

Standard thrombophilia testing may miss these rare variants. Functional assays, such as thrombin generation testing and APC resistance assays, can help detect abnormal clotting responses in the absence of identifiable mutations. Additionally, whole exome and genome sequencing in patients with unexplained familial thrombosis has begun to uncover novel variants of uncertain significance (VUS), pointing toward the polygenic or oligogenic basis of thrombophilia in many individuals. The recognition of rare and novel thrombophilia variants is clinically relevant, particularly in unexplained VTE at a young age, a strong family history of thrombosis with a negative standard workup, and recurrent thrombosis despite anticoagulation.

Future research efforts should focus on large-scale genomic studies, better phenotyping, and the incorporation of polygenic risk scores to improve risk stratification. The identification of additional variants may eventually inform personalized prophylaxis and treatment strategies.

## 6. Thrombophilia Score

Technically, there is no universally recognized score that quantifies “thrombophilia” as a standalone factor. Instead, inherited or acquired thrombophilias are typically incorporated into broader thromboembolic risk assessment models or specific clinical algorithms. In practice, identifying thrombophilia (e.g., Factor V Leiden mutation, prothrombin mutation, protein C or S deficiency, antiphospholipid antibodies, etc.) often raises the overall risk of venous, and in some instances arterial, thrombosis. The contribution of individual predisposing factors to the thrombophilic state cannot be quantified, and this is one of the elements that make the creation of a score complex. Furthermore, this contribution may be subject to variability in the presence of some comorbidities and particular conditions (oncological patients, pregnancy, inflammatory states, etc.)

Nonetheless, various thromboembolic risk scores do factor in thrombophilia. For example, the Caprini Risk Assessment Model, widely used to assess VTE risk in surgical patients, awards points for age, obesity, prior thrombosis, and inherited or acquired thrombophilia. Higher totals indicate a greater VTE risk and guide prophylactic intervention. Similarly, the Padua Prediction Score, used for hospitalized medical patients, includes thrombophilia as an additional risk element [73]. In oncology, the Khorana Risk Score does not always list thrombophilia explicitly, yet its presence is typically considered in the overall clinical evaluation of thrombosis risk in cancer patients [74].

Specialized algorithms and guidelines highlight the role of thrombophilia in other scenarios as well. In obstetrics, for instance, the Royal College of Obstetricians and Gynaecologists (RCOG) outlines specific risk assessment schemes that factor in either inherited or acquired thrombophilias, thereby increasing the total risk score and potentially indicating the need for low-molecular-weight heparin prophylaxis. For secondary prevention for patients with a history of thrombotic events, predictive models such as the DASH score or the Vienna Prediction Model estimate the likelihood of recurrence, sometimes incorporating specific thrombophilias into their calculations [75,76].

In conclusion, there is no single, internationally standardized “Thrombophilia Score” that quantifies thrombophilia on its own. Rather, thrombophilia is integrated into broader thromboembolic risk scores (e.g., Caprini, Padua, and Khorana) and into specialized guidelines (such as those for pregnancy), where its presence elevates the overall risk profile and informs decisions regarding prophylaxis or anticoagulant therapy.

## 7. Promising Thrombophilia Biomarkers

As our understanding of the pathophysiology of thrombophilia continues to evolve, a growing number of biomarkers have emerged with potential diagnostic, prognostic, and therapeutic relevance. Among these, P-selectin, E-selectin, Factor VIII (FVIII), Factor XI (FXI), neutrophil extracellular traps (NETs), extracellular vesicles (EVs), and microparticles (MPs) represent particularly promising candidates that could significantly enhance our ability to detect and manage thrombotic risk in various clinical contexts.

### 7.1. P- and E-Selectin

P-selectin and E-selectin members of the selectin family mediate interactions between endothelial cells, leukocytes, and platelets. Often referred to as “endothelial” selectins, they were first identified and characterized in the mid-to-late 1980s. P-selectin was the first of the selectins to be identified. In 1984, researchers discovered this protein while studying activated platelets, using antibodies that specifically targeted platelet surface molecules [77,78]. Initial studies described P-selectin as a glycoprotein with a molecular weight of approximately 140,000, which was absent in resting platelets but became highly expressed following activation with thrombin and other mediators.

In 1985, Stenberg et al. [79] referred to this protein as granule membrane protein-140 (GMP-140) due to its localization within the α-granules of unstimulated platelets. Their findings demonstrated that GMP-140 was translocated from the granules to the plasma membrane upon platelet activation. Around the same time, Berman et al. [80] provided additional evidence for this protein’s role in platelet activation and named it platelet activation-dependent granule-external membrane protein (PADGEM).

By 1989, further studies revealed that P-selectin was present in platelets and endothelial cells, where it was stored in specialized intracellular granules known as Weibel–Palade bodies. Some authors showed that endothelial P-selectin could rapidly mobilize to the cell surface upon stimulation by histamine, thrombin, C5b-9, and other inflammatory mediators [81,82,83]. This discovery underscored the protein’s broader role in vascular inflammation and immune responses.

That same year, the cDNA encoding P-selectin was cloned, and a sequence analysis revealed that it was a cysteine-rich protein closely related to another newly identified endothelial adhesion molecule, ELAM-1 (endothelial leukocyte adhesion molecule-1). E-selectin, originally named ELAM-1 (endothelial leukocyte adhesion molecule-1), was identified in 1986. Unlike P-selectin, which is stored in granules and rapidly mobilized upon activation, E-selectin is not pre-formed but is synthesized de novo in endothelial cells in response to pro-inflammatory cytokines, such as IL-1 and TNF-α [84].

Bevilacqua et al. [85] were among the first to characterize ELAM-1 as an endothelial adhesion molecule in recruiting neutrophils to sites of inflammation. Its structure and function were similar to P-selectin, further supporting the idea that these molecules belonged to a new family of adhesion proteins. The cloning of the ELAM-1 gene in 1989 confirmed its role in leukocyte–endothelial interactions and led to its renaming as E-selectin. E-selectin is a key regulator of thrombus formation and fibrin content, primarily expressed by activated endothelial cells. It facilitates thrombosis by modulating neutrophil and monocyte activity and acts in association with P-selectin to recruit leukocytes to sites of inflammation.

For these reasons, P- and E-selectin have been investigated as potential biomarkers for thrombosis. The soluble form of P-selectin (sP-selectin) is derived primarily from the proteolytic cleavage of transmembrane P-selectin, which is shed from activated platelets and endothelial cells. sP-selectin has been evaluated in several studies as a biomarker for VTE. Several authors have reported elevated P-selectin levels in patients with DVT [86]. Additionally, Papalambrosm et al. showed a substantial decline in P-selectin levels after seven days of heparin therapy in DVT patients, suggesting its potential role in monitoring treatment response [87].

Other studies have explored the diagnostic value of combining P-selectin with other biomarkers, such as D-dimer, and clinical scoring systems like the Wells Score. Ramacciotti et al. showed that P-selectin combined with a Wells Score of ≥2 had a specificity of 96% and a positive predictive value of 100% for confirming DVT. Conversely, a P-selectin < 60 ng/mL, coupled with a Wells Score <2, effectively ruled out DVT with a sensitivity of 99% and a negative predictive value of 96% [74]. Similar findings were found by Vandy et al. [88].

Evidence from animal studies showed that inhibiting P-selectin can influence atherosclerosis progression, fibrin deposition, and thrombus development [89].

In conclusion, P-selectin is a valuable emerging biomarker for venous thrombosis. Its noninvasive nature, coupled with a high positive predictive value, makes it a useful tool for guiding early management. Furthermore, in combination with other diagnostic tests, P-selectin holds promise as a target for future risk assessment and therapeutic strategies in thrombotic disease management. While P-selectin appears within six hours of DVT, E-selectin is upregulated later, approximately two days after thrombosis onset [90]. Studies in murine models have shown that E-selectin deficiency leads to a reduced thrombus burden, lower fibrin content, and decreased vein wall inflammation and fibrosis. It enhances neutrophil recruitment through interactions with CD18 Macrophage-1 antigen (Mac-1) and sLex, facilitating leukocyte adhesion and extravasation into the thrombus and surrounding tissue [91]. Some studies found that E-selectin is implicated in VTE and post-thrombotic syndrome (PTS) [92,93]. However, conflicting studies suggest that E-selectin may not be as effective a biomarker for DVT as sP-selectin, as it remains primarily endothelial-bound and is less readily released into the circulation. Thus, while E-selectin plays a critical role in thrombosis and inflammation, its utility as a circulating biomarker remains debated.

### 7.2. FVIII

FVIII is a sialoglycoprotein with an essential role in normal hemostasis by acting as a cofactor for FIX to accelerate thrombin generation. While FVIII deficiency leads to bleeding disorders, such as hemophilia A, elevated FVIII levels are a recognized risk factor for thrombophilia, contributing to both venous and arterial thrombotic events. Studies have shown that individuals with FVIII levels above the 90th percentile (≥150 IU/dL) have a two- to fivefold increased risk of VTE compared to those with normal levels [94,95]. Moreover, persistently elevated FVIII levels after an initial thrombotic event are associated with a higher likelihood of recurrent thrombosis, reinforcing FVIII’s role in thrombophilia [96]. FVIII levels are influenced by both genetic and environmental factors [83]. Specifically, FV Leiden and the FIIG20210A mutation are often associated with elevated FVIII levels, compounding the thrombotic risk [96].

Some individuals inherit naturally high FVIII levels without other thrombophilic mutations, suggesting a genetic predisposition to FVIII overexpression. Non-O blood group individuals (A, B, or AB) have higher FVIII levels due to the reduced clearance by von Willebrand factor (vWF), leading to an increased risk of VTE compared to those in blood group O [97,98,99,100]. Acquired factors influencing FVIII include inflammatory conditions, such as rheumatoid arthritis, inflammatory bowel disease, and chronic infections, which can lead to sustained FVIII elevation, promoting thrombophilia; endothelial activation and injury, seen in conditions like diabetes, hypertension, and atherosclerosis, can stimulate FVIII production and release, contributing to a hypercoagulable state; liver disease, while severe liver dysfunction reduces FVIII synthesis, mild liver disease can lead to a relative imbalance in coagulation factor production, sometimes resulting in elevated FVIII levels; and cancer, particularly hematologic cancers, can drive increased FVIII synthesis as part of the prothrombotic cancer-associated thrombophilia [101,102].

A critical factor in thrombophilia assessment is the FVIII/vWF ratio [103]. Normally, FVIII and vWF rise proportionally. However, in thrombophilic states, FVIII may be disproportionately elevated, leading to an increased FVIII/vWF ratio [104]. A high FVIII/vWF ratio suggests excessive FVIII production or reduced clearance, both of which contribute to thrombosis risk. Non-O blood group individuals exhibit a different FVIII/vWF dynamic due to variations in vWF clearance, which can further impact thrombotic risk [105].

The COVID-19 pandemic provided critical insights into FVIII’s role in thrombophilia. Severe COVID-19 is characterized by a hypercoagulable state, with significantly elevated FVIII and vWF levels, contributing to widespread microvascular and macrovascular thrombosis [106].

Elevated FVIII in COVID-19 patients was associated with an increased risk of VTE, including pulmonary embolism and microvascular thrombosis [107]. FVIII was found to be a key component of COVID-19-associated coagulopathy, linking inflammation and thrombosis. Persistently high FVIII levels post-COVID were observed in some patients, suggesting a long-term prothrombotic state, emphasizing the need for extended thromboprophylaxis in high-risk individuals [108].

In conclusion, FVIII is a crucial biomarker in thrombophilia, with elevated levels strongly linked to an increased risk of VTE. Its variability due to age, genetic predisposition, and inflammatory states underscores its complexity in thrombotic disorders. The FVIII/vWF ratio provides additional insight into FVIII dysregulation, and recent findings from COVID-19 have further highlighted FVIII’s role in hypercoagulability. Given its significance in thrombophilia, FVIII assessments should be integrated into thrombosis risk evaluations and long-term management strategies.

### 7.3. FXI

FXI is a serine protease synthesized by the liver that circulates in the blood in its inactive form. It has a typical dimeric structure, consisting of two identical 80 kDa subunits that share a significant homology with prekallikrein. FXI is converted into its active form, FXIa, by FXIIa in the presence of high-molecular-weight kininogen on negatively charged surfaces. Alternatively, FXI can also be activated independently of FXIIa by thrombin or FXIa itself. As a key component of the intrinsic coagulation pathway, also referred to as the “contact phase” of coagulation, FXI contributes primarily to thrombin generation, thereby playing a crucial role in hemostasis. The activation of FXI by thrombin acts as an amplification mechanism to produce additional thrombin. Thrombin, in turn, has two main procoagulant functions: (a) stabilizing clot formation by converting fibrinogen to fibrin and (b) inhibiting fibrinolysis by activating thrombin-activatable fibrinolysis inhibitor (TAFI), a carboxypeptidase that removes C-terminal lysine residues, which serve as binding sites for tissue plasminogen activator (tPA) and plasminogen on fibrin [109]. Notably, the role of FXI in coagulation may extend beyond thrombin activation. Puy C et al. showed that FXIa may enhance FXa and thrombin production by inhibiting the tissue factor pathway inhibitor in platelets and endothelial cells [110].

While FXI deficiency, also known as hemophilia C, is associated with a bleeding tendency, elevated FXI levels are associated with an increased risk of thrombotic events, including DVT, PE, and ischemic stroke.

Mechanistically, FXI levels enhance fibrin formation, reduce fibrinolysis, and promote clot stability, thereby contributing to a prothrombotic state. Genetic variations in the F11 gene have also been implicated in altered FXI activity, further supporting its role in thrombophilia [111].

A pivotal study published in the *New England Journal of Medicine* demonstrated that individuals with FXI levels above the 90th percentile had a 2.2-fold increased risk of DVT compared to those with lower levels [112]. This association remained significant even after adjusting for other risk factors, indicating that high FXI levels independently contribute to thrombotic risk. Then, several authors confirmed these findings. A case–control study showed that patients with persistently elevated FXI levels had a 5.2-fold increased risk of DVT. This study highlighted that not only elevated but also persistently high FXI levels significantly amplify the risk of venous thrombosis.

The biological mechanism underlying this association involves FXI’s role in thrombin generation and fibrinolysis inhibition. By promoting thrombin generation, increased FXI levels enhance fibrin formation and stabilize clots, thereby increasing the propensity for thrombosis.

Thus, measuring FXI levels can be valuable in assessing thrombotic risk, particularly in individuals with a history of unexplained thrombotic events or in those with other predisposing factors. However, it is essential to consider that FXI levels can vary over time and may be influenced by acute phase reactions. Therefore, multiple measurements and comprehensive clinical evaluation are recommended to accurately interpret FXI levels in the context of thrombophilia assessment.

### 7.4. Neutrophil Extracellular Traps

Neutrophil extracellular traps (NETs) are web-like structures consisting of decondensed chromatin, histones, and antimicrobial proteins released by neutrophils in response to various stimuli, such as infection, inflammation, and activated platelets [113]. This process, known as NETosis, represents a unique defense mechanism designed to trap and eliminate pathogens. However, excessive or dysregulated NET formation has been implicated in a range of pathological conditions, including thrombosis. Recent studies have identified neutrophils and neutrophil extracellular traps (NETs) as common components in human arteriovenous thrombi, mouse deep vein thrombosis (DVT), and other thrombotic disease models [114,115,116,117].

NETs contribute to thrombosis through multiple mechanisms, including (i) platelet activation and aggregation: NETs provide a scaffold that enhances platelet adhesion and activation, promoting clot formation; (ii) TF expression: NET-associated proteins can upregulate TF, a key initiator of the coagulation cascade; (iii) endothelial damage: NETs induce endothelial dysfunction, exposing procoagulant surfaces that facilitate clotting; (iv) impaired fibrinolysis: NETs protect thrombi from degradation by inhibiting fibrinolysis, thereby promoting clot persistence [118].

The overall effect of NETs on thrombosis is evidenced by their ability to promote erythrocyte-rich thrombus formation in vitro, with electron microscopy confirming the direct binding of erythrocytes to NETs. Additionally, NETs interact with fibronectin and vWF to facilitate platelet adhesion and activation [119,120]. Their association with fibrinogen further enhances fibrin deposition, increasing thrombus stability. Consequently, thrombi containing NETs exhibit a greater sensitivity to tPA compared to fibrin-dominant thrombi [121]. Experimental studies using DNase I in mouse models have demonstrated that NET degradation effectively prevents intravascular microthrombosis, indicating a significant role of NETs in thrombosis [122,123]. However, Noubouossie et al. [118] suggest that the prothrombotic effects of NETs are primarily mediated by their DNA and histone components rather than the NET structures themselves, indicating the need for further investigation.

Studies have also shown that activated platelets can stimulate NET formation and release through mechanisms involving P-selectin and high-mobility group box protein 1 [123,124]. This suggests a reciprocal relationship between NETosis and thrombosis, where their interplay contributes to a self-perpetuating cycle. This process may be particularly relevant in conditions such as thrombophilia or increased thrombotic risk following infection.

Specific NET markers, such as citrullinated histone H3 (CitH3), neutrophil elastase (NE), histones, and the MPO-DNA complex, can serve as biomarkers for diagnosing and predicting the prognosis of thrombosis-related conditions. Evidence from experimental and clinical studies supports the role of NETs in thrombosis. Indeed, increased levels of NET markers have been described in VTE patients [125,126,127,128,129]. However, their specificity in reflecting NET formation remains uncertain. While these biomarkers correlate with thrombosis severity and hypercoagulability, their ability to specifically reflect NETs-driven thrombosis remains uncertain, particularly in cancer and COVID-19 patients. First, NET biomarkers are commonly measured in serum and plasma, though these are not the direct sites of NET formation. Citrullinated histones, often used as NET markers, have also been detected in apoptosis and other non-NET-related conditions. Similarly, NE is not essential for NET formation, as its deficiency does not prevent NET release. MPO, another commonly used marker, is also expressed in monocytes and macrophages, while nucleosomes may originate from lymphocytes, red blood cells, or tumor cells. Extracellular DNA, often considered a NET biomarker, can also be released during apoptosis, necrosis, pyroptosis, or other active secretion processes. Due to the low specificity of individual NET biomarkers, combining two or more markers may provide a more reliable assessment of NET formation. Commonly measured markers, such as H3Cit, MPO-DNA complexes, NE, and nucleosomes, are typically analyzed using the enzyme-linked immunosorbent assay (ELISA), while DNA is quantified via polymerase chain reactions (PCRs) or fluorometric assays. However, variations in sample types (plasma vs. serum), preanalytical processing (e.g., collection time, processing temperature, and centrifugation conditions), and assay methodologies contribute to inconsistencies across studies. Differences in antibodies, assay techniques, detection instruments, and manufacturers lead to inconsistencies in results. Notably, the specificity of an ELISA for detecting certain NET biomarkers, such as MPO-DNA complexes, remains questionable. Thus, robust, standardized, and highly specific assays are needed to ensure accurate and reproducible results before drawing solid clinical conclusions.

Additionally, efforts to develop quantitative formulas for NET markers and their levels in circulation or local lesions could enhance diagnostic precision. Improving the sensitivity of NET detection methods may further contribute to advancements in precision medicine and outcome predictions.

However, inconsistencies in existing studies highlight the need for stronger experimental evidence to confirm the impact of NET inhibition on thrombolysis and thrombosis prevention. Additionally, concerns regarding potential immunosuppression and an increased risk of bleeding require further investigation. Future research on NETs is expected to provide innovative strategies and methodologies for addressing thrombotic diseases.

Most studies rely on case–control or cross-sectional designs comparing thrombotic patients to healthy controls. However, cohort studies tracking thrombotic events over time within the same population would provide stronger evidence for the diagnostic and predictive value of NET biomarkers. The routine clinical detection of NET biomarkers for thrombosis is premature without more robust data. The reliability of NET biomarker findings may be affected by concurrent infections, inflammation, the use of anticoagulants, antiplatelet therapy, cancer treatments, and invasive procedures. Future well-designed studies should aim to determine whether changes in NET biomarkers are a cause or a consequence of thrombosis by analyzing blood samples collected both before and after thrombotic events.

In conclusion, while NET biomarkers are linked to thrombosis, their specificity, reliability, and clinical applicability require further investigation. The standardization of detection methods, improved study designs, and the clarification of the causal relationship between NETs and thrombosis are essential before these biomarkers can be used routinely in clinical practice.

### 7.5. Extracellular Vesicles and Microparticles

Among the emerging biomarkers contributing to thrombophilia, extracellular vesicles (EVs) and microparticles (MPs) have garnered significant attention. During cell activation or apoptosis, these submicron-sized vesicles are released from various cell types, including platelets, endothelial cells, monocytes, and erythrocytes.

Extracellular vesicles (EVs) are heterogeneous vesicles classified into exosomes (<150 nm), microparticles/microvesicles (150 nm–1 μm), and apoptotic bodies (>1 μm). EVs facilitate cross-talk between platelets, endothelial cells, and leukocytes, promoting platelet activation, endothelial dysfunction, and the release of inflammatory mediators, all of which can enhance thrombus formation. EVs can transfer microRNAs and other genetic materials to recipient cells, modulating gene expression and potentially influencing pathways related to coagulation and inflammation. Elevated levels of EVs have been observed in patients with VTE and pulmonary hypertension (PH), correlating with disease severity [130].

Microparticles (MPs), also called microvesicles, are shed from the plasma membrane of cells undergoing activation or apoptosis and are enriched with phosphatidylserine (PS), tissue factor (TF) and various procoagulant molecules.

Several cellular sources contribute to the pool of MPs in circulation, each with unique implications in thrombophilia. Platelet-derived MPs (PMPs) are the most abundant MPs in circulation, PMPs enhance thrombin generation and contribute to clot formation by exposing phosphatidylserine and binding coagulation factors. Endothelial-derived MPs (EMPs) are indicative of endothelial dysfunction and injury, playing a crucial role in the hypercoagulability associated with thrombophilia. Monocyte-derived MPs (MMPs) express tissue factor, the primary initiator of coagulation, thereby linking inflammation and thrombosis. Finally, erythrocyte-derived MPs (RMPs) contribute to thrombotic risk in patients with hemolytic disorders or sickle cell disease. MPs exert prothrombotic activity through different mechanisms. MPs serve as a procoagulant surface by presenting phosphatidylserine, which facilitates the assembly of coagulation factor complexes, accelerating thrombin generation. MPs, particularly those from monocytes and endothelial cells, express tissue factor, a critical initiator of the extrinsic coagulation cascade. PMPs enhance platelet adhesion and aggregation, promoting thrombus formation, while EMPs contribute to vascular dysfunction by inducing inflammation and coagulation, leading to thrombophilic conditions. Notably, MPs act as carriers of pro-inflammatory cytokines, contributing to systemic inflammation and endothelial activation, further exacerbating the prothrombotic state. Individuals carrying prothrombotic mutations, such as Factor V Leiden or prothrombin G20210A, have been found to possess higher levels of TF-positive MPs, suggesting a link between genetic predisposition and MP release.

Additionally, MPs have been proposed as accessible biomarkers for hypercoagulable states and thrombosis risk in cancer patients, aiding in the identification of individuals at a higher risk for thrombotic events [131].

Overall, EVs and MPs hold significant promise as biomarkers in thrombophilia, offering insights into the underlying mechanisms of hypercoagulability and potential avenues for personalized medicine. However, challenges remain, including the need for standardized methodologies for EV/MP isolation and characterization, as well as large-scale studies to validate their clinical utility. The commonly used laboratory tests for EVs include flow cytometry, nanoparticle tracking analysis (NTA), transmission electron microscopy (TEM), Western blotting and ELISAs, high-resolution flow cytometry, and imaging flow cytometry. Key Challenges in EV/MV laboratory analysis include the pre-analytical variability, sample type (plasma vs. serum), collection tubes, centrifugation protocols, and freeze–thaw cycles, which all impact the EV concentration and integrity. Additionally, the lack of harmonized protocols complicates inter-study comparisons. There is no universally accepted gold standard for EV/MV measurements. Reference particles and calibration beads are often used but do not fully replicate EV behavior in biological samples. Additionally, preparations are prone to contamination by lipoproteins, protein aggregates, and cellular debris, especially in ultracentrifugation-based isolation. Quantifying EVs accurately remains difficult due to their small size, heterogeneity, and the limitations of current detection platforms. Discriminating between EV subtypes based solely on size or common surface markers is insufficient.

While multiple laboratory methods are available to analyze extracellular vesicles, none are yet optimal for routine clinical use. The field is evolving toward improved standardization, multimodal approaches (combining size, marker expression, and function), and international guidelines (e.g., MISEV 2018 by the International Society for Extracellular Vesicles). Greater efforts in validation, reference standard development, and interlaboratory reproducibility will be key to unlocking EVs’ full diagnostic and prognostic potential.

### 7.6. Future Potential of Promising Thrombophilia Biomarkers

The future landscape of thrombophilia diagnosis and management is poised for a significant transformation driven by emerging biomarkers. These biomarkers offer an opportunity to move beyond the limitations of traditional thrombophilia testing and toward a more dynamic, real-time understanding of thrombotic risks influenced by inflammatory, immunologic, and cellular pathways.

One of the most promising future applications is the individualized risk stratification of thrombosis. Unlike genetic tests that assess static risk, these emerging biomarkers reflect dynamic, context-dependent processes such as endothelial activation, inflammation, and coagulation system perturbations. Incorporating biomarkers like soluble P- and E-selectin or NET-derived components into clinical risk scores could enable tailored anticoagulation strategies, especially in complex scenarios such as cancer-associated thrombosis, autoimmune disorders, and post-surgical care.

Another exciting avenue is using these biomarkers to monitor disease activity and treatment response. For instance, tracking levels of NETs or procoagulant EVs could help assess the effectiveness of anti-inflammatory or anticoagulant therapies. Similarly, FVIII and FXI levels may guide the anticoagulation duration or intensity in patients with idiopathic or recurrent VTE. This can significantly reduce undertreatment and overtreatment, minimizing bleeding risks while maintaining thrombotic protection.

Several of these biomarkers are indicators and active mediators of thrombosis, opening doors for targeted therapeutic development. FXI inhibitors are already under investigation in clinical trials, and interventions aimed at blocking selectins or degrading NETs are in preclinical or early clinical phases. Identifying patients with elevated levels of these targets could allow for biomarker-guided therapy selection, furthering the principles of precision medicine in thrombosis care.

As technologies advance, integrating thrombophilia biomarkers into multi-omics platforms (e.g., proteomics, transcriptomics, and metabolomics) and machine learning models will likely enhance their predictive power. Such approaches reveal complex patterns and interactions that single markers might miss, potentially uncovering new biomarker combinations with superior prognostic value. These tools can also help identify currently unrecognized subtypes of thrombophilia, supporting more nuanced classification and management strategies.

The use of circulating biomarkers may also expand into screening asymptomatic individuals with high thrombotic risk, such as those with a family history of cancer or autoimmune diseases. Although routine screening remains controversial, developing low-cost, high-throughput assays for these markers could make targeted screening more feasible.

Several hurdles must be addressed for these biomarkers to achieve widespread clinical use. These include standardizing assays, establishing reference ranges, interlaboratory reproducibility, and cost-effectiveness validation. Collaborative international efforts and large prospective cohort studies will be critical in defining their roles within clinical guidelines and practice.

In summary, the ongoing exploration and validation of thrombophilia biomarkers such as P- and E-selectin, FVIII, FXI, NETs, EVs, and MPs hold immense promise to revolutionize how thrombotic risk is assessed, monitored, and treated. Their integration into personalized medicine, supported by advances in analytical techniques and computational modeling, could mark a paradigm shift from static risk models to real-time, individualized thrombosis management.

## 8. Arterial Thrombosis: A Clinical Challenge

Hereditary thrombophilias are classically associated with venous thromboembolism (VTE), while their role in arterial thrombosis remains uncertain and highly debated. The underlying pathophysiological mechanisms of venous and arterial thrombosis are fundamentally different: venous thrombosis is primarily driven by hypercoagulability, whereas arterial thrombosis is typically triggered by atherosclerotic plaque rupture and subsequent platelet activation [132]. Consequently, the presence of hereditary thrombophilias, such as the Factor V Leiden mutation or the prothrombin G20210A mutation, does not appear to significantly increase the risk of arterial thrombotic events. Although some studies have suggested a possible association in specific patient subgroups, definitive evidence remains lacking [133,134].

The current body of literature on this subject is limited and often inconclusive. Studies investigating the relationship between thrombophilias and arterial thrombosis have yielded conflicting results, partly due to the challenge of isolating the impact of thrombophilias from that of traditional cardiovascular risk factors, including hypertension, diabetes, hyperlipidemia, and smoking, which are the primary determinants of arterial thrombosis. Some reports suggest that thrombophilias may play a role in certain clinical scenarios, such as early-onset myocardial infarction or cryptogenic stroke in young individuals, yet the absence of large, well-controlled studies precludes definitive conclusions [134].

A notable exception is antiphospholipid syndrome (APS), an acquired thrombophilic disorder that predisposes individuals to both venous and arterial thrombosis, with a well-established association with ischemic stroke and myocardial infarction. However, unlike hereditary thrombophilias, APS involves a more complex pro-inflammatory and prothrombotic mechanism, characterized by endothelial dysfunction and platelet activation, which are central to the pathogenesis of arterial thrombosis [133].

Given these considerations, the utility of thrombophilia testing in patients with arterial thrombotic events remains controversial. Current guidelines do not recommend routine screening for hereditary thrombophilias in individuals with myocardial infarction or ischemic stroke unless specific clinical features, such as a strong personal or family history of venous thrombosis, early-onset arterial thrombosis, or the absence of conventional cardiovascular risk factors, are present. Overdiagnosis and inappropriate management are significant concerns, and clinical attention should remain focused on identifying and modifying traditional cardiovascular risk factors rather than pursuing thrombophilia testing in settings where its clinical relevance remains uncertain. Furthermore, in light of the current evidence, no single thrombophilia marker has been definitively linked to arterial thrombosis. Unlike venous thromboembolism, where specific genetic and acquired markers can help stratify risk, arterial thrombotic events arise from a multifactorial interplay between endothelial injury, platelet activation, and systemic inflammation. The heterogeneity of the arterial thrombosis pathophysiology further complicates the identification of a universal thrombophilia marker with significant predictive value. While certain markers, such as the lupus anticoagulant or antiphospholipid antibodies, have demonstrated a stronger correlation with arterial thrombosis, the same cannot be said for most inherited thrombophilic traits. Therefore, the absence of a well-defined thrombophilia profile for arterial events reinforces the need for a cautious and evidence-based approach to testing, ensuring that diagnostic strategies remain clinically meaningful and do not lead to unnecessary interventions.

## 9. Conclusions

Thrombophilia remains a complex clinical entity where over-testing or ill-timed testing can lead to unnecessary costs, patient anxiety, and potential mismanagement.

Beyond the clinical implications, thrombophilia testing may impose a significant psychological burden on patients. The identification of a thrombophilic defect, particularly in the absence of a personal thrombotic history or clear implications for clinical management, can provoke heightened anxiety, an altered risk perception, and psychological distress. Patients may overestimate the prognostic significance of test results, potentially resulting in unnecessary behavioral modifications or reproductive concerns. Furthermore, the knowledge of carrying a heritable condition may elicit concern for family members, amplifying emotional strain. These effects are compounded by potential financial burdens associated with testing, particularly when insurance coverage is limited or absent. Accordingly, the decision to initiate thrombophilia testing should be carefully weighed, ideally within the framework of shared decision-making, and accompanied by pre-and post-test counseling to mitigate potential adverse psychological outcomes and promote informed, patient-centered care.

Current guidelines across major health organizations increasingly advocate for a targeted, context-specific evaluation, one that considers personal and family history, the presence of clearly defined risk factors, and the potential to meaningfully change clinical management. While circulating biomarkers such as D-dimer, antithrombin, protein C, and protein S offer insights into coagulation dynamics, their interpretation requires caution due to numerous confounding variables and the risk of false positives or negatives. Genetic markers like FV Leiden and FII G20210A play a definitive role in certain patient subsets but lack a sufficient prevalence to justify routine population-wide screening. In moving toward personalized thrombophilia diagnostics, clinicians must balance the implications of positive or negative test results against the patient’s broader clinical profile. This approach ensures that testing guides relevant therapeutic decisions, such as the duration or intensity of anticoagulation, and supports preventive strategies, particularly in populations at higher risk. By adhering to consensus recommendations and integrating evidence-based criteria, healthcare providers can optimize patient outcomes, reduce the burden of unnecessary testing, and maintain a vigilant yet judicious stance on thrombophilia assessment.

## Data Availability

Not applicable.
